# From capacity-building to capacity-bridging: Indigenous Research Liaisons as a strategic intervention to bridge Indigenous communities’ capacities in the British Columbia health research environment

**DOI:** 10.1177/11771801251409186

**Published:** 2026-02-10

**Authors:** Krista Stelkia, Tara Lise Erb, Lindsay Botterill, Julianne Cecile Barry

**Affiliations:** 1 Faculty of Health Sciences, Simon Fraser University, Canada; 2 Poche Centre for Indigenous Health, Faculty of Medicine and Health, The University of Sydney, Australia; 3 Centre for Collaborative Action on Indigenous Health Governance, Simon Fraser University, Canada; 4 British Columbia Network Environment for Indigenous Health Research, Simon Fraser University, Canada

**Keywords:** capacity-bridging, capacity-building, Indigenous health research network, Indigenous health research, Indigenous Research Liaison, self-determination

## Abstract

The British Columbia Network Environment for Indigenous Health Research (BC NEIHR) supports research capacity, engagement, and leadership of Indigenous communities, collectives, and organizations (ICCOs) in Indigenous-led health research. To support Indigenous self-determination in the health research landscape, the BC NEIHR centers Indigenous approaches in research policy and practice. The BC NEIHR uses the term capacity-bridging, opposed to capacity-building, to acknowledge and support existing ICCO research capacities, distinct cultural knowledges, and community-led perspectives. The BC NEIHR enacts its commitment to ICCO self-determined health research through capacity-bridging initiatives including the Indigenous Research Liaison (IRL) program. We share how the IRL program created meaningful change through ICCO relationship-building, knowledge sharing and resource development, health research partnerships, and institutional systems-level change. The IRL program represents a case study of how an organizational-level strategic intervention can help shape and inform the transformation of research environments to better support Indigenous self-determination in Indigenous-led health research.

## Introduction

In Canada, research involving Indigenous peoples has a long-standing history of being extractive, paternalistic, and conducted without consideration of the needs and priorities of Indigenous peoples and communities directly (McPhail-Bell et al., 2018; Smith, 2021). The research landscape has been predominantly established using a Western approach to knowledge production that limits Indigenous perspectives and worldviews (Erb & Stelkia, 2023). For example, research administrative structures and policies fail to consider the distinct needs, cultures, and ways of knowing of Indigenous peoples (Kilian et al., 2019; Strengthening Indigenous Research Capacity, 2021). Common research administrative barriers include a lack of training and capacities relevant to working with Indigenous peoples (Gittelsohn et al., 2020), limitations affecting administrators’ ability to support Indigenous research (Brunette-Debassige, 2023), and Indigenous communities, collectives, and organizations (ICCOs) ineligibility to hold and administer research funds from mainstream research funders (Erb & Stelkia, 2023). Due to structural and systemic barriers, distrust of institutions, and lack of respect for Indigenous ways of knowing, Indigenous communities have not received significant support for self-determined research (Erb & Stelkia, 2023; Hart, 2010; Schnarch, 2004).

Distrust of Western research stems from extensive colonial research experiences that perpetually disregarded community ethics, consent, and relevance (Anderson, 2019; Smallwood et al., 2021). Racist institutions and policies, such as the residential school system, were not only tools of oppression and control, but used for unethical and harmful research conducted by non-Indigenous researchers (Hyett et al., 2018; Mosby, 2013). As highlighted by Mosby (2013), Indigenous bodies were perceived as subjects for experiments, and residential schools as laboratories. This dehumanizing approach voided individual autonomy and consent and actively silenced Indigenous peoples’ self-determination. Nutrition experiments, lacking ethical rigor of informed consent, forced malnutrition of Indigenous children attending residential schools. These experiments resulted in recommendations, dictated by Western ‘experts’, for mainstream Canadian nutritional education and diets, which were not reflective or relevant to Indigenous cultures and ways of life (Mosby, 2013).

Further examples of exploitative research included the Nuu-chah-nulth peoples’ experiences with non-consensual use of blood samples. Blood samples collected from community members within a study on arthritis at the University of British Columbia, Canada, were taken without community consent by the lead researcher to the United States and further to the United Kingdom, where the samples were used and shared for unrelated research with no direct benefit to Nuu-chah-nulth peoples (Dalton, 2002). These examples, two of many, demonstrate that despite ongoing efforts to decolonize health practices, colonial perspectives are still deeply embedded within systemic processes of Western mainstream research environments (Steel, 2013). Beyond such violations, current research continues to be deficits-based, failing to recognize the distinct strengths of Indigenous ways of knowing and being (Hyett et al., 2019; Stelkia et al., 2023; Thiessen et al., 2020). These negative research practices highlight a critical need to center ethical engagement and approaches in Indigenous health research to promote self-determination of Indigenous peoples in the research environment.

## British Columbia Network Environment for Indigenous Health Research

The British Columbia Network Environment for Indigenous Health Research (BC NEIHR) is part of the national Network Environment for Indigenous Health Research (NEIHR) program, which includes nine provincial and regional Indigenous-led networks and one national coordinating center. The NEIHR program aims to address the needs and priorities of Indigenous peoples in health research in Canada (Canadian Institutes of Health Research, 2018). The BC NEIHR supports and facilitates Indigenous-led health research that is woven from values, knowledge systems, protocols, priorities, and leadership of ICCOs and Indigenous researchers, students, and trainees in BC. As of November 2024, the BC NEIHR had 405 registered members, including ICCOs (80), Indigenous students and trainees (149), Indigenous academic and health professionals (88), and allied academic and health professionals (88). A majority of members, 73%, were studying or employed in Indigenous health research.

## From capacity-building to capacity-bridging

To advance Indigenous-led health research, the BC NEIHR supports the concept of capacity-bridging instead of capacity-building. Although widely used, the term capacity-building has been perceived as paternalistic through its reference to non-Indigenous individuals ‘teaching’ Indigenous peoples about research. Capacity-bridging, developed within the Visioning Health project (Canadian Aboriginal AIDS Network [CAAN], n.d.), more accurately describes the reality that ICCOs already possess, and are not required to be taught, research capacities including how to plan, organize, operationalize, and lead. Instead, ICCOs require opportunities and support to bridge existing capacities into the context of research (CAAN, n.d.; Erb & Stelkia, 2023). This idea of ‘bridging’ capacities lies within an ethical space, described by Indigenous scholar Willie Ermine (2007), where research values, acknowledges, and is grounded in Indigenous worldviews, knowledge systems, and practices. Within this ethical space, non-Indigenous researchers who understand the fundamentals of healthy relationships and equity have an appropriate environment to transfer relational capacities to their research. Capacity-bridging shifts away from the Western notions of paternalism or saviourism and instead upholds Indigenous capacities to create space for additional growth, and supports communities beyond funding by cultivating reciprocal, respectful, and reflective relationships.

## Capacity-bridging supports Indigenous self-determined research

Capacity-bridging promotes Indigenous self-determination in research by shifting decision-making power from Western researchers and systems to ICCOs and Indigenous peoples. Self-determination, the individual and collective right to sovereignty over all decisions regarding health, education, political, and economic systems (United Nations General Assembly, 2007), is a critical factor impacting the overall health and wellness of individuals and communities (Auger, 2016; Halseth & Murdock, 2020; Reading & Wien, 2009). Within research, the BC NEIHR views self-determination as the right of Indigenous peoples to determine their own research priorities, have equitable access to funding and support, and have Indigenous ethics and protocols respected.

Through capacity-bridging, the BC NEIHR acknowledges, mobilizes, and supports existing capacities, cultural knowledges, and perspectives of ICCOs, rather than assuming the requirement to teach or build capacity ‘from a zero base’ (Erb & Stelkia, 2023; McPhail-Bell et al., 2018, p. 2). Capacity-bridging advances Indigenous leadership in health research, creating space for ICCOs to identify, explore, and address their own health priorities within their diverse cultural, social, political, and economic contexts (Auger, 2016; Cooper & Driedger, 2018). Capacity-building, within Western paternalistic approaches, overlooks the strengths, perspectives, and capacities of ICCOs and Indigenous peoples, diminishing autonomy and benefits for the community.

## Indigenous Research Liaison (IRL) program: a strategic intervention for capacity-bridging

The BC NEIHR enacts its commitment to supporting ICCOs self-determination in health research through capacity-bridging initiatives, including the development and implementation of the IRL program. Launched in April 2020, the intention of the program is for IRLs to provide direct, community-level research support to ICCOs within BC. The program employs up to five IRLs, representative of each BC health region – Fraser Health, Interior Health, Island Health, Northern Health, and Vancouver Coastal Health. IRLs typically hold an undergraduate degree and have experience in health research, knowledge of Indigenous worldviews, and experience working with ICCOs. To date, IRLs have been Indigenous, primarily graduate students from research-based programs, and employed between 6 months and 3 years. IRLs are provided comprehensive training informed by ICCOs needs and priorities, and ethics and cultural safety courses, and receive ongoing guidance from Elders, Knowledge Holders, and BC NEIHR research team and staff. The IRL program follows a distinction-based approach to adapt and respect diverse ICCOs’ priorities and capacities. Support and accountability occur through frequent communication, regular team meetings, and monthly reporting.

The IRL program, funded through the BC NEIHR program funding, provides complementary support – free of charge – to ICCOs. ICCOs include First Nations communities, Métis chartered communities, urban Aboriginal Friendship Centres, or independent First Nation, Métis, or Inuit organizations or collectives. ICCOs research teams range in size and capacity from teams of a few volunteers to teams with existing partnerships with academic researchers or organizations. Each distinct ICCO has varying degrees of research capacity, ranging from initial interest in developing their own projects and relationships, but may not have developed capacity to administer their own research and funding, to ICCOs with higher capacity that have more research infrastructure such as staff, resources, governance, and terms of reference to administer their own research and funding. The IRL program supports ICCOs across BC and ICCOs funded through the BC NEIHR. ICCOs funding programs are 1-year terms; however, ICCOs often remain engaged in the network and participate in gatherings and capacity-bridging activities.

IRLs, referred to by ICCOs as ‘people on the ground’, play a critical role in developing, facilitating, and promoting Indigenous-led health research within BC through supporting ICCOs’ success in major research funding competitions (Erb & Stelkia, 2023). Within their role, IRLs support ICCOs to determine funding opportunities, connect with academic and other partners, complete applications for BC NEIHR and other external research funding, and, within funding applications, develop budgets and communicate ideas and knowledge sharing and mobilization strategies. A key component of the IRL program and a crucial aspect of capacity-bridging (Erb & Stelkia, 2023; Redman-MacLaren et al., 2012) is cultivating respectful and reciprocal relationships that recognize existing ICCO strengths and capacities. IRLs act as a bridge between ICCOs and institutional and academic research environments to provide capacity-bridging support and opportunities for ICCOs to plan, organize, operationalize, and lead research.

The evaluation of the IRL program follows a wholistic framework. Western methods of evaluation are hierarchical, binary, based on outcomes, and focus on the ‘what’. Indigenous ways of knowing are relational and value both the outcomes and the process, including the reciprocity built throughout (Wilson, 2008), offering a perspective on the ‘how’ (Goodchild, 2022). Unlike the ‘what’, the ‘how’ provides insight into relations, interactions, and movements within systems. The BC NEIHR evaluation framework (Erb & Loppie, 2022) moves beyond the individual to acknowledge the interconnectedness and relations between the cosmos, humans, Land, other-than-humans, and the spiritual realm (Goodchild, 2022; Kovach, 2021). The IRL program indicators are not rooted solely in quantitative success – for example, the number of successful ICCOs applications – but also based on relationality and reciprocity. Within this approach, IRLs, ICCOs, and collaborators are not evaluated but instead evaluation includes the results of what was done and what could be done better, together.

The IRL program is a strategic intervention developed by the BC NEIHR to enact capacity-bridging initiatives to support ICCOs self-determined health research. The aim of this article is to share four components of the IRL program that support Indigenous self-determination in Indigenous-led health research. The four components of the IRL program include (1) ICCOs relationship-building, (2) knowledge sharing and resource development, (3) health research partnerships, and (4) institutional systems-level change. While outlined separately for clarity, these components are not mutually exclusive and often interconnect and overlap.

### ICCO relationship-building

The first component of the IRL program is relationship-building, which aims to create meaningful and ongoing research relationships. Relationship-building, including IRL outreach and capacity-bridging activities, is actively documented by each IRL. Monthly reports are critical for accountability, reflection, and to inform successes, challenges, and spaces for growth in relationship-building. During the first 4 years of the program, IRLs connected with approximately 300 ICCOs, totaling over 700 engagement and outreach interactions via email, phone, and Zoom.

Relationship-building is demonstrated through the role of IRLs in the BC NEIHR-funding model. Annual BC NEIHR funding available for ICCOs is intended for ICCOs to center Indigenous-led health research development and knowledge sharing and mobilization. IRLs seek out interested ICCOs and provide support throughout the entire application process. The IRL program’s first phase included reaching out to ICCOs to introduce the BC NEIHR and its purpose. Through dedicating significant time and resources, IRLs’ outreach ranged from phone calls to in-person connections through visits to ICCOs and ICCO attendance at BC NEIHR-hosted events. The journey of relationship-building requires a significant investment in time by IRLs to establish and strengthen relationships that are needed before starting and throughout the work with ICCOs.

To build genuine relationships, IRLs take direction from ICCOs with respect to ICCO capacity and trust. Relationships, unique to each ICCO, can take several years to build, demonstrating the importance of time required to develop and foster meaningful and trusting relationships. Within these relationships, ICCOs regularly invite IRLs to participate in and help plan ICCO events. For example, an IRL was invited to a canoe launching and celebration dinner for National Indigenous Peoples Day. Other examples include an IRL being invited to a naming ceremony, which is a very sacred and ‘closed’ ceremony, and another was invited to assist and participate in planning a Nation’s powwow. These invitations highlight the ways in which IRLs create genuine and trusting connections with ICCOs that foster meaningful and supportive partnerships. Given the continuous mistrust of ICCOs toward research, receiving invitations to ICCO events demonstrates the strengthening of reciprocity through successful IRL relationship-building.

### Knowledge sharing and resource development

The second component of the IRL program is knowledge sharing and resource development. To support ICCOs, IRLs developed accessible resources – distributed and available online to ICCOs – including nano-tutorials and step-by-step guides for the BC NEIHR-funding application, an interactive map of ICCO ethics frameworks in BC, research and funding resources, and podcasts (Erb & Stelkia, 2023). To reach a wider audience, and to engage in alternative methods of knowledge sharing, IRLs developed a BC NEIHR podcast entitled ‘Research DE-Colonized: Ethical, Indigenous-Led Health and Wellness Research in Canada’ (https://researchdecolonized.buzzsprout.com/). The podcast connects with communities, scholars, and health professionals to highlight stories of decolonizing Indigenous health and wellness research. Topics include journeys into Indigenous health and wellness research, findings from Indigenous-led health studies, research funding and development, and ethical research protocols when working with Indigenous peoples and ICCOs.

Knowledge sharing and resource development is guided by ICCOs. Through continuous engagement with ICCOs, IRLs respond by creating resources reflective of ICCO needs and priorities. Examples of resources included modules on research interviewing, research development strategies, grant writing, and land-based methodologies. Knowledge sharing is further supported by in-person events that enable networking opportunities and space to build connections between the BC NEIHR, ICCOs, Indigenous students and trainees, and Indigenous community members.

### Health research partnerships

The third component of the IRL program is health research partnerships. The BC NEIHR aims to cultivate a decolonized health research environment, built on respect and reciprocity, that nurtures Indigenous capacity and advances Indigenous self-determination. To cultivate this decolonized environment, it is not only critical to build relationships with ICCOs but also with non-Indigenous health organizations and institutions. IRLs seek out partnerships with health systems, research institutes, and health organizations. Through these partnerships, IRLs actively support Indigenous self-determination in research through policy change, advocacy, and sharing lessons learned. These strategic partnerships create space and opportunities needed within health systems to transform the health research environment. For example, through collaboration with Research Ethics BC, IRLs supported an environmental scan of BC research ethics boards to examine current practices of reviewing Indigenous research and identified existing gaps and challenges within the research ethics review process (Erb & Littlechild, 2022).

Partnerships and reciprocity established and built are indicators within the wholistic evaluation of the IRL program. Success within institutional-level partnerships includes invitations from organizations for IRLs to fill advisor and facilitator roles. IRLs provide guidance on organizational policies and initiatives, hold advisory roles on planning committees, and participate in organizational events. In addition, IRLs facilitate presentations, meetings, and training workshops on topics including research ethics and methodologies, relational leadership, and relationship-building in Indigenous health research. An indicator of the IRL program included the Indigenous Health Research Ethics Symposium, co-hosted in 2024 by the BC NEIHR and the BC SUPPORT Unit, in partnership with the Centre for Collaborative Action on Indigenous Health Governance. The gathering, held on the traditional Musqueam territory, advanced engagement and understanding of Indigenous health research ethics. IRLs supported and networked with nearly 100 attendees including ICCOs, Indigenous students and trainees, Indigenous Elders, and leadership from health authorities and organizations. The symposium is an example of cultivating a decolonized health research environment, where IRLs worked alongside non-Indigenous organizational partners to create a space that respected self-determination of ICCOs and Indigenous peoples in research. These partnerships and resulting indicators can directly impact systems-level change.

### Institutional systems-level change

The fourth and final component of the IRL program is institutional systems-level change, which is required to advance decolonizing health research environments. Through a relational approach, the IRL program provides support to ICCOs and non-Indigenous organizations, creating spaces and conditions needed for systems-level change (Goodchild, 2022). For example, IRLs prioritize Indigenous cultural safety training to promote change within the system. IRLs provide resources and facilitate presentations and workshops on cultural safety, which are co-developed by IRLs and BC NEIHR team members who have extensive experience and expertise in cultural safety. IRLs actively share input and provide guidance for integration of cultural safety within organizational structures and events. The active support provided through resources, presentations, and guidance creates opportunities for organizations to create policy change through the development of organizational cultural safety frameworks.

IRLs prioritize ICCOs and their needs through supporting changes that address institutional barriers, including research funding barriers. The BC NEIHR ensures the BC NEIHR-funding application process is approachable by shifting away from academic language and using accessible, culturally relevant, and appropriate language, providing support from IRLs throughout the process, and responding to ICCO feedback. IRLs also advocate for ICCOs within institutional funding. For example, while working with an ICCO on an external funding application, there was a shift in ICCO capacity due to staffing shortages. The supporting IRL successfully advocated on behalf of the ICCO for an extension from the external funding organization. Another example demonstrated institutional policy change, where guidance from IRLs directly led to changes in funding procedures of a new health authority funding competition.

IRLs provide guidance to ICCOs and non-Indigenous organizations for ethical guidelines for Indigenous health research. IRLs supported the creation of an interactive map of ICCO research ethical frameworks within BC, intended as a resource for ICCOs to support the development of their own ethical frameworks. To inform organizational and policy change, IRLs participated in Indigenous ethics discussions with Elders and organizational partners and organized and participated in ethics process sharing circles held for Indigenous university faculty and trainees. Further, IRLs collaborated with universities and health authorities to enhance community ethical engagement through development of Indigenous engagement plans and protocols. These examples highlight how the IRL program supported identifying and addressing existing gaps and barriers within the health research system to promote systems-level change to better meet the needs of ICCOs in health research.

## Conclusion

Through the BC NEIHR, the IRL program enacts capacity-bridging to uphold and advance Indigenous self-determination through ICCO relationship-building, knowledge sharing and resource development, health research partnerships, and institutional systems-level change. Considering the harmful impacts and resulting mistrust of Western research, Indigenous health research requires a decolonized health research environment to support the overall health and wellness of Indigenous peoples and communities. To support Indigenous-led and self-determined research, relationship-building with ICCOs must occur first. Once a relationship is established, capacity-bridging initiatives acknowledge, recognize, and bridge diverse ICCO capacities to the research environment to support ICCO self-determined research. Institutional partnerships are critical to support and strengthen institutional understandings of Indigenous ways of knowing and required to transform the research environment to be reflective of ICCO needs and priorities. The IRL program created meaningful change within ICCO-led research across the province through the relationships, partnerships, and reciprocity built. The IRL program demonstrates a strategic intervention to address institutional barriers faced by ICCOs and inform systems-level change for the transformation of Indigenous health research environments. The ongoing success of the IRL program depends on sustained research funding from the BC NEIHR, as well as the continuation of trusted relationships with ICCOs and partners. While grounded in the Canadian health research context, the IRL program offers a transferable model for other countries seeking to support Indigenous-led health research by centering Indigenous self-determination, capacity-bridging, and culturally grounded approaches.
